# Supplementary material for: An Osteoblast-Specific Enhancer and Subenhancer Cooperatively Regulate Runx2 Expression in Chondrocytes
Source: Int J Mol Sci. 2025 Feb 14;26(4):1653. doi: 10.3390/ijms26041653 (PMC11855347; doi:10.3390/ijms26041653)
Supplement: Supplementary file 1 [file ijms-26-01653-s001.zip › ijms-3423641-supplementary.pdf]

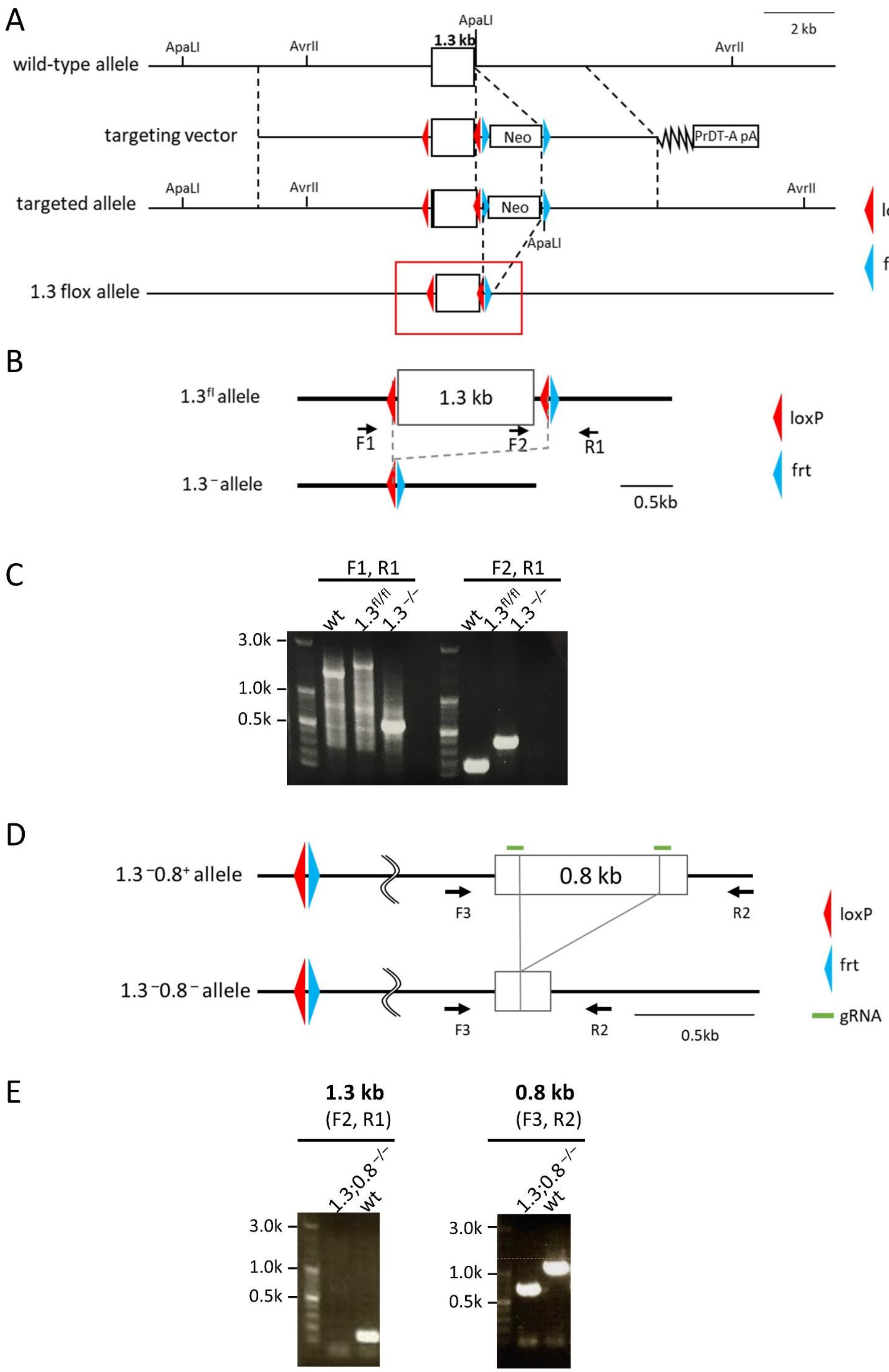


**Supplementary Figure S1.**
Generation of 1.3$^{-/-}$ mice and 1.3;0.8$^{-/-}$ mice. (A) Strategy for generating a 1.3-kb floxed mouse line. The red box is magnified in B. (B) Strategy for generating a 1.3$^{-/-}$ mouse line. (C) PCR for genotyping to detect wild-type, 1.3$^{fl/fl}$, and 1.3$^{-/-}$ mice using primer pairs F1 and R1 (wt: 1600 bp, 1.3$^{fl/fl}$ : 1974 bp, 1.3;0.8$^{-/-}$: 531 bp), and F2 and R1 (wt: 185 bp, 1.3$^{fl/fl}$ : 359 bp, 1.3;0.8$^{-/-}$: none). (D) Strategy for generating a 1.3;0.8$^{-/-}$ mouse line. (E) PCR for genotyping to detect wild-type and 1.3;0.8$^{-/-}$ mice using primer pairs F2 and R1 (wt: 185 bp, 1.3;0.8$^{-/-}$: none) and F3 and R2 (wt: 1184bp, 1.3;0.8$^{-/-}$: 723bp).

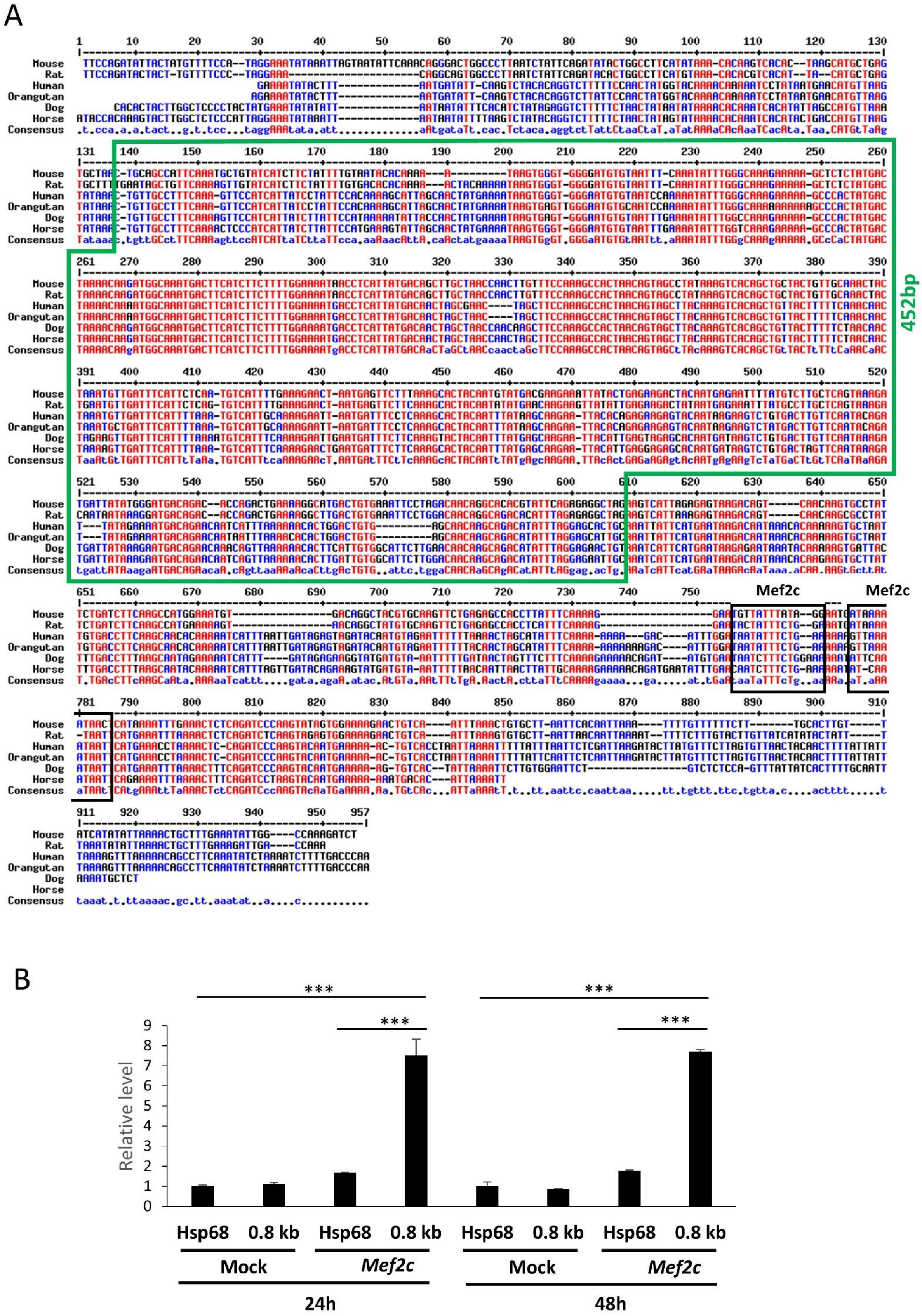


**Supplementary Figure S2.**

Homology of the 0.8-kb region and reporter assay of 0.8-kb DNA fragment. (A) Homology of the mouse, human, rat, orangutan, dog, and horse 0.8-kb regions searched by MultAlin accessed on 18 March 2016. Red letters indicate high homology. The green box shows the 452-bp region. Two Mef2c-binding motifs are shown in black boxes. (B) Reporter assays of the luciferase vectors of Hsp68 or Hsp68 with 0.8-kb DNA fragment in SW1353 cells transfected with the Mock or *Mef2c* expression vector. Data are the mean ± SE. ***$p<0.001$.

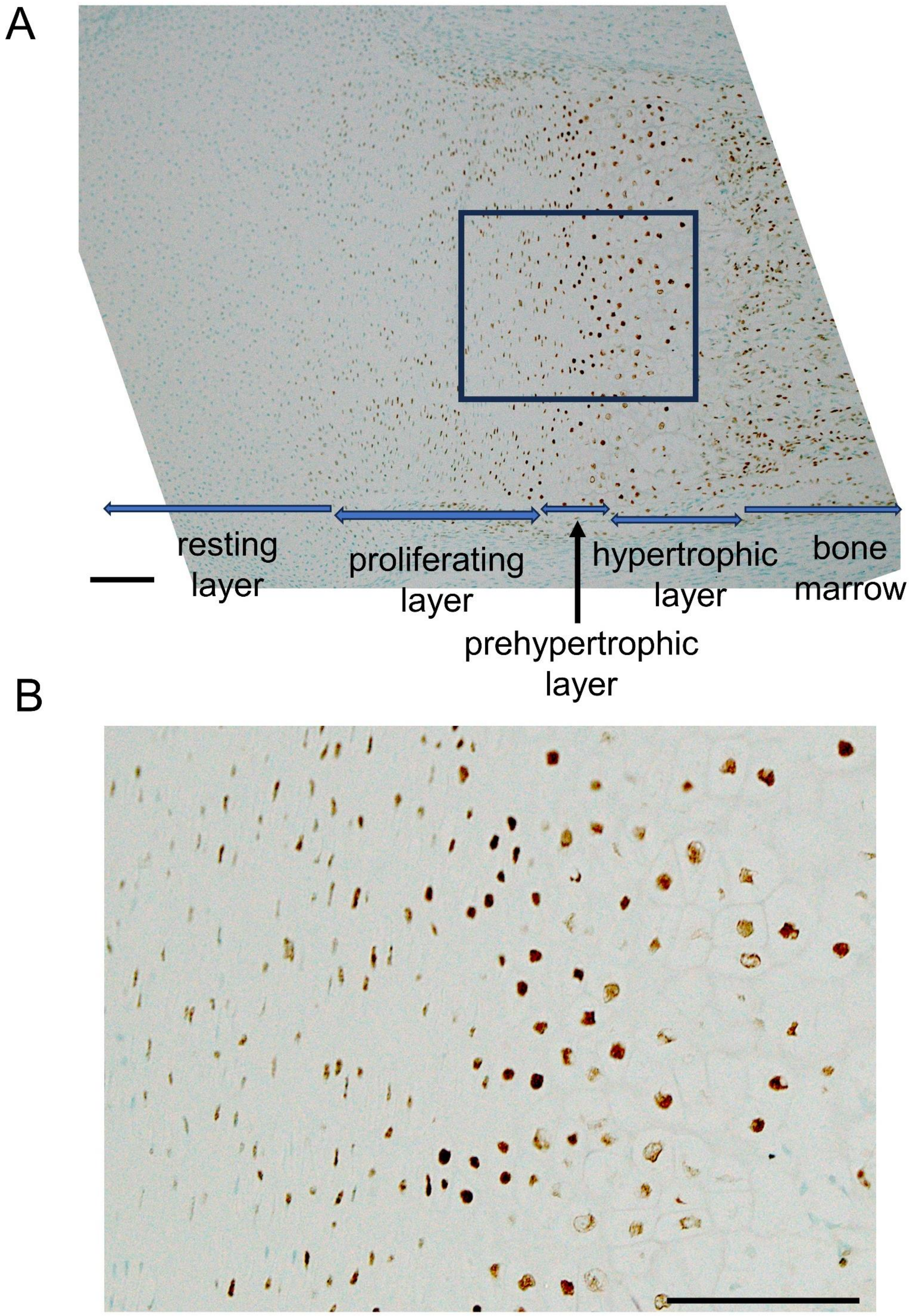


**Supplementary Figure S3.**
Immunohistochemical analysis of Runx2 expression in the newborn wild-type femur. Immunohistochemistry was performed using a monoclonal rabbit anti-Runx2 antibody (Cell Signaling) and a rabbit two-step test kit (ZSGB-BIO, Beijing, China) as the secondary antibody. The boxed region in A is magnified in B. Scale bars: 100 μm.

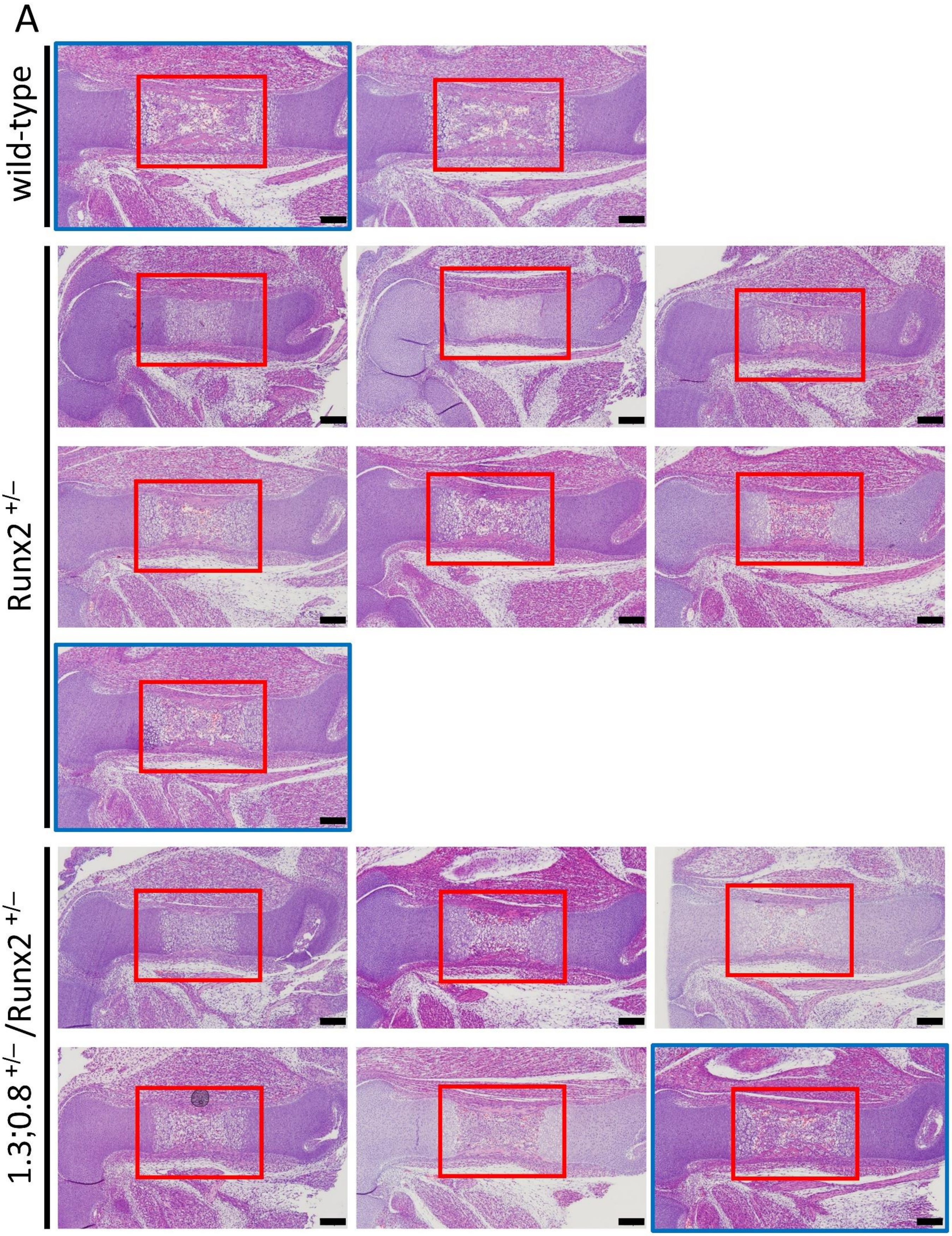


**Supplementary Figure S4A**

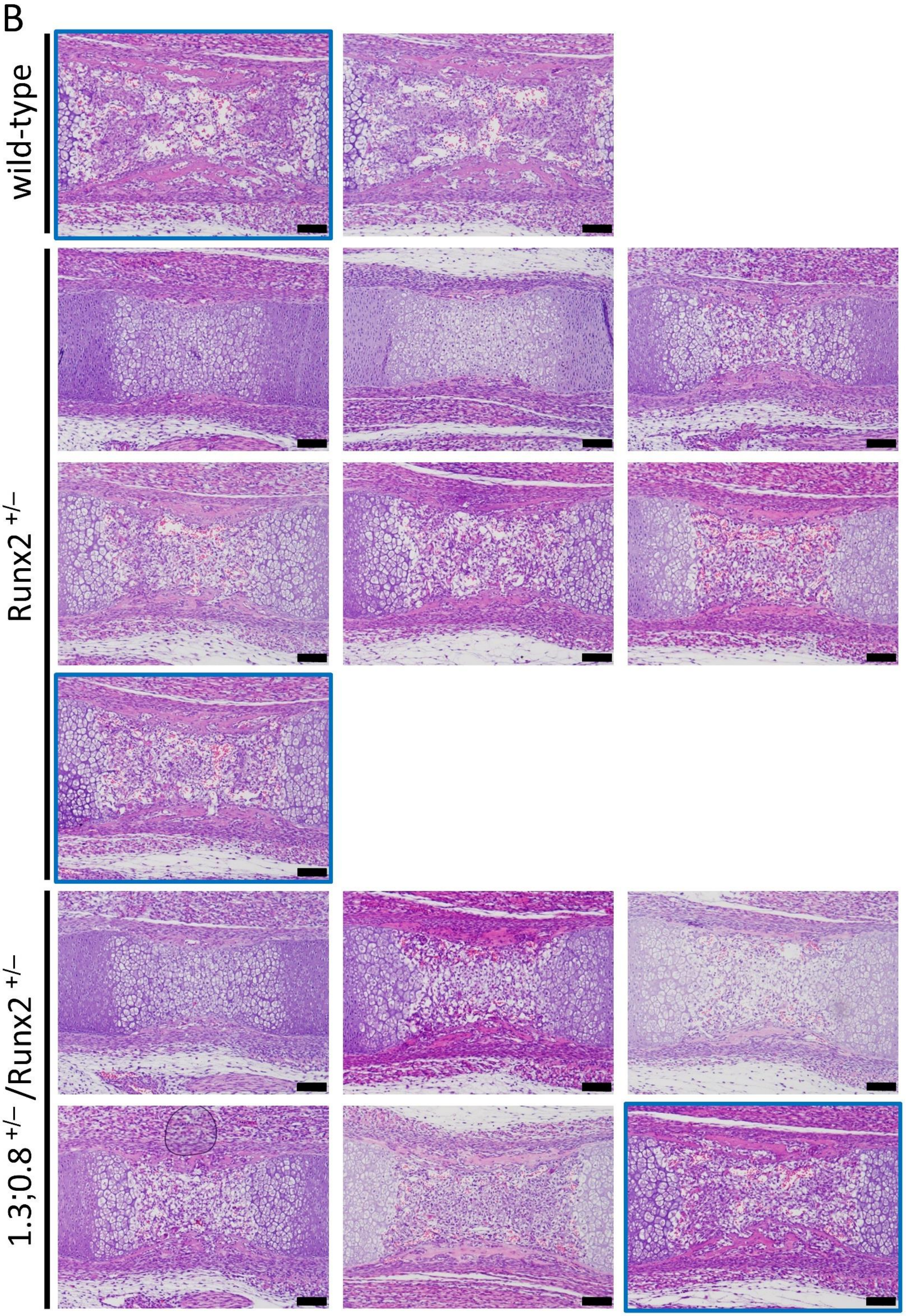


**Supplementary Figure S4.**
H-E stained sections of femurs of wild-type, *Runx2*$^{+/-}$, and 1.3;0.8$^{+/-}$/*Runx2*$^{+/-}$ embryos at E15.5. As there was a variation in the progress of endochondral ossification in *Runx2*$^{+/-}$ background, the sections are aligned according to the progress. The boxed regions in A are magnified in B. The pictures with blue frames were shown in Fig. 8H-M. Scale bar: 200 μm (A), 100 μm (B).

**Supplementary Table S1. Primer and gRNA sequence**

| Analysis | Gene or enhancer | | Primer(5'-3') |
|---|---|---|---|
| Realtime-RT-qPCR | *Actb* | F | CCACCCGCGAGCACAGCTTC |
| | | R | TTGTCGACGACCAGCGCAGC |
| | *Runx2* | F | AACAAGACCCTGCCCGTG |
| | | R | TGAAACTCTTGCCTCGTCCG |
| Genotyping | 1.3 kb | F1 | TCACAGACAGACACACAGAC |
| | | F2 | GCCCACTCTCGGTAGTTATT |
| | | R1 | ACAGCTGGCTGAAAGTTGCT |
| | 0.8 kb | F3 | GGCATTTCCAGAATAGACAGG |
| | | R2 | TTTTTCTGTCACACATAACATGAG |
| gRNA | 0.8 kb | up | TTGTAATACACAAAAATAAG |
| | | down | TCAAGCCATGGAAATGTGAC |
| ChIP | 0.8 kb | 5' | TCTATTCAGATATACTGGCCTTCA |
| | | 3' | GGAAACAAGTTGGTTAGCAAGC |
